# Systemic lupus erythematosis with severe aplastic anemia successfully treated with rituximab and antithymocyte globulin

**Published:** 2014

**Authors:** Wenbin Liu, Zhiping Hu, Shengyun Lin, Jianghua He, Yuhong Zhou

**Affiliations:** 1 Wenbin Liu, Department of Hematology, First Clinical Medical School of Zhejiang Chinese Medicine University, China.; 2 Zhiping Hu, Department of Hematology, First Clinical Medical School of Zhejiang Chinese Medicine University, China.; 3 Shengyun Lin, Department of Hematology, First Clinical Medical School of Zhejiang Chinese Medicine University, China.; 4 Jianghua He, Department of Hematology, First Clinical Medical School of Zhejiang Chinese Medicine University, China.; 5 Yuhong Zhou, Department of Hematology, First Clinical Medical School of Zhejiang Chinese Medicine University, China.

**Keywords:** Aplastic anemia, Rituximab, Antithymocyte globulin, Systmic lupus erythematosus

## Abstract

Hematologic disorders are very common in Systmic lupus erythematosus (SLE).First presentation of SLE with severe aplastic anemia (SAA) is extremely rare. We report a patient with the diagnosis of secondary SAA associated with SLE. Conventional therapy was not effective. She received Rituximab (RTX) and Antithymocyte globulin (ATG) therapy, her response was satisfactory finally. Her hematologic parameters were within normal ranges until last follow-up, eight months and six months after therapy was initiated with RTX and ATG, respectively. This is the first time RTX and ATG were successfully used in the treatment of SAA secondary to SLE.

## INTRODUCTION

Systemic lupus erythematosus (SLE) is an autoimmune disease which affects multiple tissues and organs and is commonly seen in young women. Hematologic abnormalities are commonly associated with SLE.^1^ Dysregulation of B lymphocyte cells plays a crucial role in the pathogenesis of the disease. Autoimmune thrombocytopenia (AITP) and antoimmune hemolytic anemia (AIHA) are the most common hematologic manifestations of SLE seen in 20% to 40% and 5% to 10% of patients, respectively.^2^

Acquried aplastic anemia (AA) is an immune-mediated bone marrow (BM) failure attacked by autoreactive effector T cells and BM is the main target organ, which is characterized by pancytopenia and bone marrow aplasia that is potentially life threatening. Etiological mechanism of AA secondary to SLE is not clearly defined. It is suggested that dysregulation of B and T lymphocyte cells may play important role in the destruction of hematopoietic bone marrow stem cells. Though life-threatening hematologic disturbances are infrequently reported, however, SLE with initial clinical presentation of SAA is extremely rare.

## CASE REPORT

A 25-year-old female patient presented with dizziness, canker sore, purpura and petechiae-like lesions on the lower limbs, and low-grade irregular fever for one month duration. There was no history of joint pain, rash, prior neurological disturbance, or any complaint attributable to chest, cardiovascular or abdominal systems, except photosensitivity. Physical examination revealed a normotensive, average-built, canker sore and severely pale female. There was no icterus, cyanosis, lymphadenopathy, splenohepatomegaly or pedal edema. Heart, chest, and neurological system examinations were unremarkable.

On investigations, her laboratory data results showed pancytopenia (WBC, 900/mm^3^, hemoglobin (Hb) 3.8 mg/dL, platelet count, 3,000/mm^3^, reticulocyte count, 4,000/mm^3^). Antinuclear antibody, anti-nRNP antibody and anti-histone antibody were positive; however, anti-dsDNA antibody, anti-sm antibody, anticardiolipin antibody, rheumatoid factor, Coombs test (direct and indirect) and CD55CD59 were negative. Liver and renal function tests were within normal range. The echocardiography result was normal. Bone marrow aspiration and biopsy were performed for pancytopenia which showed less than 20% cellularity, megakaryocytes were absent and fatty change, suggestive for SAA. The G-banding of 20 metaphase BM cells showed all cells to be a normal karyotype, 46, XY. She fulfilled the American College of Rheumatology criteria and the British Committee for Standards in Haematology for the diagnosis of SLE and AA, respectively.

The patient was treated with the diagnosis of secondary SAA associated with SLE.As a first step, treatment was started with the pulse dose of methylprednisolone (MP, 500mg/day×4) and high-dose immunoglobulin (IVIg,400mg/Kg/day×3) in addition to supportive care such as blood transfusion. Then, the patient has been on 10mg/d of prednisolone for maintenance therapy soon. Unfortunately, despite adding cyclosporine (CSA, 300mg/day) for 2 months to the treatment regimen, response was very poor. There was no increase in blood cell counts observed two months later.

As a next step, because of poor response to routine treatment, we tried RTX (375mg/m^2^/week×4). But before that we further improved the laboratory data results showing Foxp3+ 0.17%, CD19+ 9.47%, CD69+CD4+ 0.38% and CD69+CD8+ 4.87%. After 2 months, hematologic results were not suitable and transfusion dependency presented. (WBC, 1, 500/mm^3^; hemoglobin (Hb), 5.8 mg/dL; platelet count, 5,000/mm^3^). The percentage of different lymphocytocyte types showed some changes (Foxp3+ 0.16%, CD19+ 0.10%, CD69+CD4+ 18.2%, CD69+CD8+ 17.4%). Clinical symptoms of oral ulcers and photosensitivity were resolved. However, Antinuclear antibody, anti-nRNP antibody and anti-histone antibody were still positive.

At last choice, she was considered as a candidate for therapy with rabbit-antithymocyte globulin (ATG, 3mg/kg/day×5) which was started and the patient was closely followed.then 300mg/d CSA and granulocyte ecolon-stimulating factor(G-CSF) were continued. After 3 months, her clinical and hematological response was remarkable and eventually achieved transfusion independence (WBC, 6,500/mm^3^; hemoglobin, 7.8 mg/dL; platelet count, 16,000/mm^3^). Six months after she was discharged, her hematological parameters were within normal limits.The percentage of different lymphocytocyte types had remarkably changed (Foxp3+ 0.89%, CD19+ 1.16%, CD69+CD4+ 2.53%, CD69+CD8+ 3.47%). A BM aspiration performed at 6 months of the ATG therapy showed myeloid and erythroid hyperplasia with normal megakaryopoiesis. However, antinuclear antibody, anti-nRNP antibody and anti-histone antibody were never negative.

## DISCUSSION

Hematological complications are frequently seen in SLE, anaemia, leucopenia and thrombocytopenia always behind other manifestation of SLE. They may result from marrow failure or excessive peripheral cell destruction, all of which may be immune mediated. The pathogenesis of SLE is complex, which includes loss of immune tolerance, defective B lymphocyte cells suppression and abnormal T lymphocyte cells immune responses.^3^ Although pathophysiology of AA is very different from that of SLE, AA is in essence also an autoimmune disorder. However the exact pathogenetic link, if any, between the two diseases has not yet been clearly elucidated.

Conventional therapy increases the risk of infection and malignancies, and responses to current therapies are often incomplete, even ineffective, especially for severe SLE patients. Because of cyclophosphamide(CTX)-induced ovarian failure remains a potentially severe drawback, the patient has not received CTX therapy. The patient was unresponsive to conventional immunosuppressive therapy, such as glucocorticoid.

B lymphocytes play a crucial role in the pathogenesis of SLE, through at least three broad mechanisms. Polyclonal B lymphocyte cells produce autoantibodies, activating the complement system and immune responses. The central role for B lymphocyte cells in the pathogenesis of SLE provides the rationale for use of anti-CD20 monoclonal antibody RTX in its treatment.^4^ Alishiri et al. first described a case of AA as primary manifestation of SLE who was successfully treated with RTX.^5^ We also found the expression levels of CD19 on the residual B cells of the patient which were significantly decreased shortly after the treatment of RTX and persisited at least eight months. Although the clinical symptoms of the patient were improved through RTX therapy, hematologic disorder did not get much better. However, at the same time, the T lymphocyte cell surface molecules of CD69+CD4+ and CD69+CD8+ were upregulated, which may have exacerbated immunologic disorders.

The quality and quantity of T lymphocyte were equally important in the pathogenesis of SLE. Although the mechanism of pancytopenia in SLE is not clear, different mechanisms have been postulated. Bone marrow failure was found to be mediated by cytotoxic T lymphocytes through direct cytotoxicity via FP as pathway or indirectly through cytokines.^6^ The SLE associated with AA has also been associated with an excess of CD8+ T cell. Regulatory T cells(Tregs) are key players in the maintenance of peripheral immune tolerance through suppression of the proliferation and release of proinflammatory cytokines from immune cells.^7^ Decreased numbers of Tregs are associated with impaired immune homeostasis and development of autoimmune diseases. The transcription factors Foxp3 has key roles in Tregs development and function. whenever AA or SLE is decreased at presentation.^8^^,^^9^ We found the Foxp3 of the patient decreased remarkably, which was improved after ATG therapy, that in parallel with clinical responses and decreased expression of CD69+CD4+and CD69+CD8+.

Serological abnormalities improved always in parallel with clinical responses for SLE. However, significant alterations were not seen after RTX and ATG therapy. This may suggest that B and T lymphocytes cells, similar to conventional immunosuppression, have a greater effect on pathogenic antibodies, such as anti-dsDNA, than other autoantibodies thought to have less pathogenic potential.^10^

In conclusion, we herein presented the first case where RTX and ATG were successfully used as an alternative treatment for a patient with secondary SAA associated with SLE who was refractory to routine therapy. The high expression of CD69+CD4+ and CD69+CD8+ may be useful for predicting response to RTX and ATG therapy in secondary SAA associated with SLE. These novel treatments include B and T lymphocyte cells depleting that block the costimulatory pathways of B and T lymphocyte cells. However, there still remain many issues including a mechanism of action, an optimal dose and long-term efficacy, which need further investigation.
